# Traditional Chinese Medicine for Senile Dementia

**DOI:** 10.1155/2012/692621

**Published:** 2011-07-25

**Authors:** Zhihong Lin, Jie Gu, Jin Xiu, Tingyan Mi, Jie Dong, Jyoti Kumar Tiwari

**Affiliations:** ^1^Naturals and Bioscience, Unilever R&D Shanghai, Shanghai 200233, China; ^2^Molecular Aspects of Health, Unilever R&D Vlaardingen, Vlaardingen 3130 AC, The Netherlands

## Abstract

Traditional Chinese Medicine (TCM) has a 3000 years' history of human use. A literature survey addressing traditional evidence from human studies was done, with key result that top 10 TCM herb ingredients including *Poria cocos*, *Radix polygalae*, *Radix glycyrrhizae*, *Radix angelica sinensis*, and *Radix rehmanniae* were prioritized for highest potential benefit to dementia intervention, related to the highest frequency of use in 236 formulae collected from 29 ancient Pharmacopoeias, ancient formula books, or historical archives on ancient renowned TCM doctors, over the past 10 centuries. Based on the history of use, there was strong clinical support that *Radix polygalae* is memory improving. Pharmacological investigation also indicated that all the five ingredients mentioned above can elicit memory-improving effects *in vivo* and *in vitro* via multiple mechanisms of action, covering estrogen-like, cholinergic, antioxidant, anti-inflammatory, antiapoptotic, neurogenetic, and anti-A*β* activities. Furthermore, 11 active principles were identified, including sinapic acid, tenuifolin, isoliquiritigenin, liquiritigenin, glabridin, ferulic acid, Z-ligustilide, N-methyl-beta-carboline-3-carboxamide, coniferyl ferulate and 11-angeloylsenkyunolide F, and catalpol. It can be concluded that TCM has a potential for complementary and alternative role in treating senile dementia. The scientific evidence is being continuously mined to back up the traditional medical wisdom.

## 1. Introduction

Cognitive impairment or dementia in elderly is associated with many disorders [1]. Alzheimer's disease (AD) is the principal type of dementia and represents about 70% of the dementia patients.

 The pathologic hallmarks of AD are senile plaques, neurofibrillary tangles, dystrophic neurites, and neuronal loss. The development of AD may be due to the improper biochemical processing of amyloid precursor protein (APP) leading to subsequent accumulation of *β*-amyloid (A*β*). The amyloid and tangle cascade hypothesis is the dominant explanation for the pathogenesis of AD [2]. Other relevant factors, including cholinergic dysfunction [3], neuroinflammation [4, 5], oxidative stress [6], and disturbance of neuronal plasticity [7], age-related loss of sex hormones [8, 9], are important and contribute to the understanding of AD pathology. 

 The 2nd most common form of dementia is vascular dementia (VD) or multi-infarct dementia, which accounts for about 15% of dementia cases [10, 11]. VD may follow after a succession of acute cerebrovascular events or, less commonly, a single major stroke. The compromised cerebrovascular circulation causes ischemia that leads to damage of the brain structure, for example, formation of white matter lesions or silent brain infarctions. VD is often related to the loss of fine motor control besides memory impairment. 

 Currently, there is no effective treatment for AD, although many treatment strategies exist [12]. Clinically, cholinesterase inhibitors (ChEIs) and N-methyl-D-aspartate (NMDA) receptor antagonists are first-line pharmacotherapy for mild-to-moderate AD, with high nonresponse rate 50–75% [13]. 

Lots of folk plants in traditional medicine are being used in age-related brain disorders for improvement of memory and cognitive function [14–16]. In China, a number of herb ingredients known from Traditional Chinese Medicine (TCM) have a long history of use for mental health. In this study, we exploited the empirically driven TCM lore and surveyed scientific data to back up the cognitive benefits, claimed by TCM.

## 2. Ancient Records on TCM for Cognitive Decline

The term “senile dementia” refers to a clinical syndrome seen in the elderly characterized by impairment of memory and cognition. So in a search of the ancient literature of TCM, the etiology, pathogenesis, and treatment for “dementia or amnesia” have been used for the survey in detail. 

### 2.1. Etiology and Pathogenesis

#### 2.1.1. Deficiency of Energy

Deficiency of energy is similar to “*Qi*” deficiency in TCM. According TCM lore Qi is the essential substance that makes up the body and maintains various physiological activities, similar to flow of energy in the body. The energy is mainly from the kidney, heart, and spleen, especially from the kidney. In TCM, the energy from the kidney is called kidney essence which can produce marrow including cerebral marrow, spinal cord, and bone marrow. The cerebral marrow can nourish the brain and maintain the physiological functions of the brain. If the kidney essence is insufficient, the production of cerebral marrow will be reduced, leading to various symptoms, such as headache, dizziness, amnesia, and retard response [17].

#### 2.1.2. Blood Stasis

Normally the blood is pumped by the heart to flow in the vessels. If blood circulation is stagnated or slowed down by certain factors such as cold, emotional disorder, aging, consumptive disease, and overstrain, it will result in retention of blood flow in the vessels or organs, a pathological condition named blood stasis. The cognitive function will decline, due to long-term global hypo-perfusion in cerebral blood flow or acute focal stroke in memory-related cerebral parenchyma [17].

#### 2.1.3. Toxin

As the function of internal organs in the elderly decline, the balance between host defense and external toxins in the body is disrupted. Pathological or physiological products occur and form toxin including waste of “water” and “endogenous fire”, which result from the poor digestion, accumulates into phlegm and retention of fluid, and caused by mental disorder, attack from pathological factors, and imbalance within the body, respectively. If such toxins can not be eliminated quickly the blood circulation and mental acuity will be affected, eventually contributing to the onset of dementia.

### 2.2. Therapy of TCM

TCM has a long history for preventing and treating cognitive decline. Although AD is a modern disease entity and has no direct analogue in the ancient Chinese medicine literature, disorders of memory and cognitive deficit are referred to throughout the classical literature. For example, in *Sheng Nong Ben Cao Jing* (Han dynasty, 1-2 century), the earliest pharmacopeia existing on materia medica in China, some TCM ingredients such as Yuan Zhi (Thinleaf milkwort), Ren Shen (Ginseng), Huang Lian (Golden thread), and Long Yan (Longan) were recorded to ameliorate the decline of people's memory. 

 In this study, 27 ancient TCM books were selected, which could be divided into 3 types, namely, Pharmacopoeias, formulae monographs and renowned TCM doctor's case studies. 

 A database was established to determine the frequency of herbs in these documents. Totally 236 formulae for improving cognitive function were identified among 27 books mentioned above (Table 1); 139 herbs were gathered from those 236 formulae and 10 TCM herbs were prioritized due to the highest frequency over 50 times (Table 2). 

 According to specification documented in Chinese Pharmacopeia [18], (i) *Poria cocos* is a diuretic with capacity to invigorate spleen function and calm the mind. Clinically, it is applicable for memory decline due to spleen deficiency and phlegm blockage; (ii) *Radix polygalae *is able to anchor the mind and eliminate the phlegm, and indicated in forgetfulness and insomnia; (iii) *Radix glycyrrhizae* is a *qi* tonic to invigorate the stomach and spleen, resolve phlegm, and clear away heat and toxin; (iv) *Radix Angelica sinensis*, as a vital blood tonic and antithrombotic agent, is especially used to treat stroke and poststroke vascular dementia induced by blood stasis; (v) *Radix rehmanniae* is another tonic used to reinforce kidney essence and marrow. Because of functionality to invigorate the energy, activate blood circulation, or eliminate the toxin, these herbs can be prescribed along or combined to exhibit a good therapeutic effect for senile dementia, for example, Zhi Ling Tang [19].

## 3. Evidence-Based Efficacy of TCM Herbs on Cognitive Decline

### 3.1. Poria cocos

*Poria cocos* (Chinese name: Fu Ling) is the dried sclerotium of the fungus, *Poria cocos* (Schw.) Wolf (Fam. Polyporaceae). 

#### 3.1.1. Functionality/Efficacy

There is suggestive evidence that *P. cocos* is memory improving regardless of absence of available clinical reports. Pharmacological research exhibited that the water extract of *P. cocos* enhanced hippocampal long-term potentiation (LTP) and improved scopolamine-induced spatial memory impairment in rats ([20, 21], Table 3).

#### 3.1.2. Mechanism of Action

Its cognitive action has been ascribed to slight cholinesterase (ChE) or acetylcholinesterase (AChE) inhibition and bidirectional regulation on cytosolic free calcium ([22–24], Table 3).

#### 3.1.3. Active Principles

The responsible actives for the cognitive benefits are unclear for the time being. Triterpene acids and polysaccharides are principal constituents of *P. cocos*, responsible for diverse bioactivities, including antitumor, anti-inflammatory, nematicidal, antioxidant, antirejection, antiemetic effects, as inhibitors against DNA topoisomerases, phospholipase A2. Besides, lecithin and choline present in the fungus are beneficially nutritional substance [25–29].

### 3.2. Radix polygalae

*Radix polygalae *is the root *Polygala tenuifolia *Willd. or *P. sibirica* L. (Fam. Polygalaceae), used as a cardiotonic and cerebrotonic, sedative and tranquilliser, and for amnesia, neuritis, and insomnia [30, 31]. 

#### 3.2.1. Functionality/Efficacy

There is strong support that thinleaf milkwort root is memory improving. BT-11, the extract of dried root of *Radix polygalae*, was developed in Korea as a functional diet with cognitive enhancing activity. Elderly with subjective memory impairment and mild cognitive impairment ascend with oral BT-11 at 300 mg/d for 4–8 weeks. Except for mild dyspepsia, no adverse events were reported [32, 33].

#### 3.2.2. Mechanism of Action

A number of investigations also sustained that *Radix polygalae *extracts functioned to promote neuronal proliferation and neurite outgrowth in normal brain and improve memory impaired by scopolamine, stress, nucleus basalis magnocellularis-lesioning operation *via* a variety of molecular pathways, including increasing glucose utilization and inhibiting AChE activity. Besides nootropic effects, *Radix polygalae *extracts protected neurons against insults induced by NMDA, glutamate, and A*β* ([34–39], Table 4(a)). In addition, anti-inflammatory activity probably contributed to the cognitive and neuroprotective efficacy, as *Radix polygalae *extracts inhibited interleukin-1 (IL-1)-mediated tumour necrosis factor (TNF)-*α* secretion, and ethanol-induced IL-1 secretion by astrocytes [40, 41].

#### 3.2.3. Active Principles

Phytochemically, *Radix polygalae *mainly contains a variety of active constituents, including saponins, xanthones, and acylated oligiosaccharides [42–44]. 

**Figure 1:**
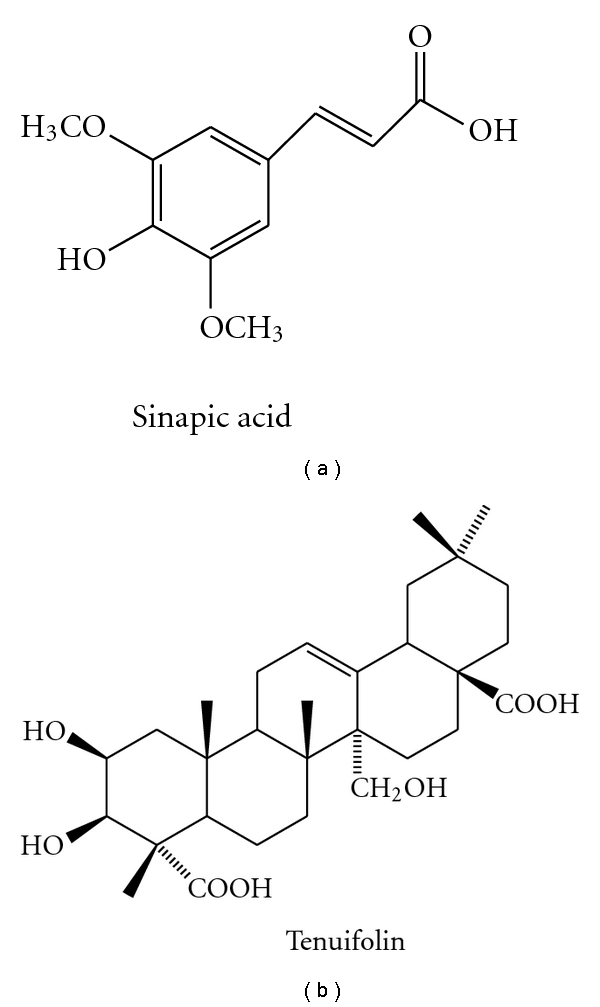
Chemical structures of sinapic acid and tenuifolin.

Saponins, especially tenuifolin isolated from tenuigenin might reinforce cognitive performance in aged and dysmnesia mice, via elevating levels of dopamine (DA) and norepinephrine (NE), and inhibiting AChE activity (Figure 1). Meanwhile, onjisaponin indicated cytoprotective activity in PC12 cells, exposed to serum deficiency or glutamate. In addition, tenuigenin facilitated memory in rats, damaged by A*β* 1–40 or ibotenic acid, via enhancing cholinergic function, or inhibiting A*β* secretion ([45–48], Table 4(b)). 

Few phytochemical principles have been isolated and identified as CNS active components. Besides tenuifolin, sinapic acid [49], a common moiety of tenuifoliside B and 3, 6′-disinapoylsucrose, reversed memory deficit induced by scopolamine and basal forebrain lesion (Table 4(b), Figure 1).

### 3.3. Radix et Rhizoma Glycyrrhizae

*Radix et rhizoma glycyrrhizae *is the dried root and rhizome, generally derived from a different plant species, with similar properties, including *Glycyrrhiza uralensis* Fisch., *G. inflata *Bat., or *G. glabra* L. (Fam. Leguminosae). 

#### 3.3.1. Functionality/Efficacy

The extracts of *Radix glycyrrhizae *reversed the cognitive deficits induced by diazepam, scopolamine, and beta-amyloid peptide 25–35 in mice at doses of 75, 150, and 300 mg/kg per oral, or diet containing either 0.5 or 1% extract, through anti-AChE and antioxidant activities. In addition, roasted licorice extracts elicited neuroprotection against brain damage after transient forebrain ischemia in Mongolian gerbils, behind which antioxidant activity was also implicated, for example, maintaining superoxide dismutase (SOD)1 level in hippocampal CA1 pyramidal cells ([50–54], Table 5).

#### 3.3.2. Mechanism of Action and Active Principles

*Radix glycyrrhizae *contains glycyrrhizin, glycyrrhizic acid, glabridin and derivatives, glabrol, glabrene, 17*β*-hydroxysteroid dehydrogenase, glucoliquiritin apioside, prenyllicoflavone A, shinflavone, shinpterocarpin, 1-methoxyphaseollin, salicylic acid, and derivatives, as well as other saponins, flavonoid glycosides, and flavonoids. 

**Figure 2:**
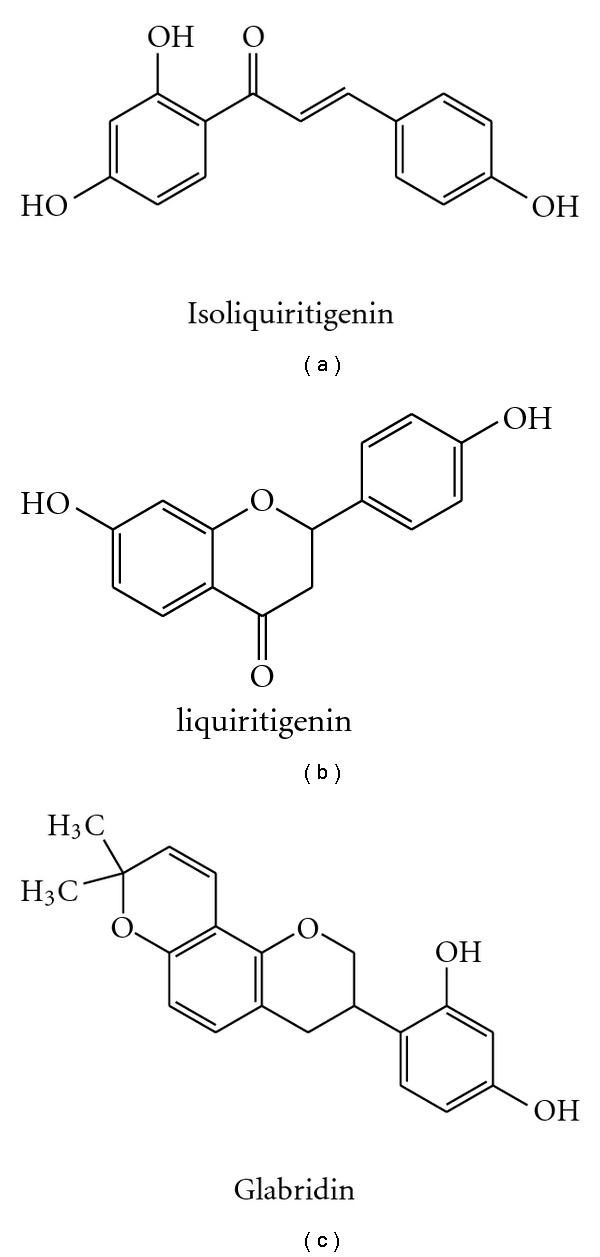
Chemical structures of isoliquiritigenin, liquiritigenin, and glabridin.

 Isoliquiritigenin, liquiritigenin, and glabridin have been identified from the *Radix glycyrrhizae* to be possible bioactive compounds ([55–58], Table 5, Figure 2).

1. Isoliquiritigenin also has the protective potential against transient middle cerebral artery occlusion-induced focal cerebral ischemia in rats, at the doses of 5, 10, and 20 mg/kg. Its protection may be attributed to amelioration of cerebral energy metabolism and antioxidant property.
2. Liquiritigenin, a plant-derived highly selective estrogen receptor *β* agonist has been identified to alleviate the cognitive recession in the elders.
3. Glabridin appears to be an active isoflavone as it improved learning and memory in mice at 1, 2, and 4 mg/kg, through targeting at ChE. Glabridin had a protective effect on cerebral ischemia injury, and neuron insult induced by staurosporine at 5, 25 mg/kg (i.p). Its underlying mechanism is probably linked to antioxidant and antiapoptotic activity.
4. Glabrene also could be beneficial to memory due to estrogen-like activities, like isoliquiritigenin, liquiritigenin, and glabridin [59–61].

### 3.4. Radix Angelica sinensis

*Radix Angelica sinensis* (Chinese: Danggui, Dong quai, Donggui; Korean Danggwi), is the dried root of *Angelica sinensis* (Oliv.) Diels (Umbelliferae).

#### 3.4.1. Functionality/Efficacy

Behaviour test displayed that *Radix Angelica sinensis* extracts ameliorated scopolamine and cycloheximide, but not p-chloroamphetamine-induced amnesia at 1 g/kg bw. In addition *in vitro* study showed that *Radix Angelica sinensis* extracts prevented the neurotoxicity induced by A**β**in Neuro 2A cells, at the doses ranging 25–200 *μ*g/mL, through antioxidant pathway ([62, 63], Table 6(a)). Furthermore, estrogenic activity of *Angelica sinensis* will probably help alleviate peri- or postmenopausal symptoms including cognitive decline in women [64, 65].

#### 3.4.2. Mechanism of Action and Active Principles

**Figure 3:**
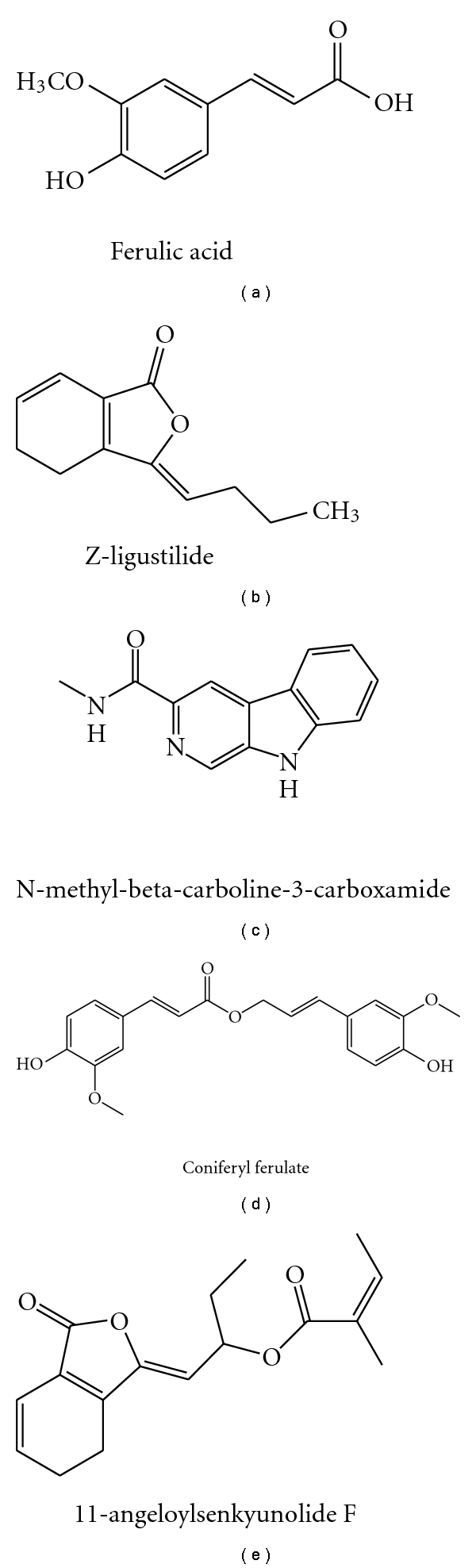
Chemical structures of ferulic acid, Z-ligustilide, N-methyl-beta-carboline-3-carboxamide, coniferyl ferulate, and 11-angeloylsenkyunolide F.

1. Ferulic acid has been identified to be an active principle because it may reverse memory deficits induced by a variety of toxins, including dl-buthionine-(S,R)-sulfoximine, trimethyltin, glutamate, A*β*1-42, scopolamine, and cycloheximide. Multiple mechanisms are probably implicated into its cognitive benefits, including inhibition on oxidative stress, activation of ChAT or enhance the cholinergic activities, competitive N-methyl-D-aspartate (NMDA) receptor antagonism, suppression on immunoreactivities of the astrocyte, and facilitation of cerebral blood flow ([66–70], Table 6(b), Figure 3).
2. Z-ligustilide has been identified to be another active component from volatile of *Radix Angelica sinensis. * It may protect brain and cognition especially against focal and global ischemia induced by permanent common carotid arteries occlusion (CCAO) and transient middle cerebral artery occlusion (MCAO) [71–73], (Table 6(b), Figure 3).
3. Additionally, N-methyl-beta-carboline-3-carboxamide, Coniferyl ferulate, and 11-angeloylsenkyunolide F were identified to be anti-AD components probably by inhibiting A*β*1-40 induced toxicity and AChE activity ([62, 74], Figure 3).

### 3.5. Radix rehmanniae

*Radix rehmanniae* is the roots of *Rehmannia glutinosa *Libosch., family *Scrophulariaceae*. 

#### 3.5.1. Functionality/Efficacy

There have been growing evidences that *Radix rehmanniae *extract possesses significant neuroprotective activity ([75, 76], Table 7). 

#### 3.5.2. Mechanism of Action

*Radix rehmanniae* extract improved learning and memory in rats with Monosodium-glutamate-(MSG-) injured thalamic arcuate nucleus at 4.5, and 9.0 g/kg, through adjusting glutamates and *γ*-amiobutyic acid (GABA) levels, as well as increasing the expression of hippocampal c-fos, nerve growth factor (NGF), NMDA receptor 1, and GABA receptor. Moreover, Rehmannia extract stimulated glial cell-derived neurotrophic factor (GDNF) gene expression in C6 glioblastoma cells, through upregulating cPKC and ERK 1/2 pathways ([76, 77], Table 7).

#### 3.5.3. Active Components

Catalpol, an iridoid glycoside, was isolated from the fresh *Radix rehmanniae*. It exists broadly in many plants all over the world and has many biological functions such as anti-inflammation, promoting of sex hormones production, protection of liver damage, and reduction of elevated blood sugar. 

**Figure 4:**
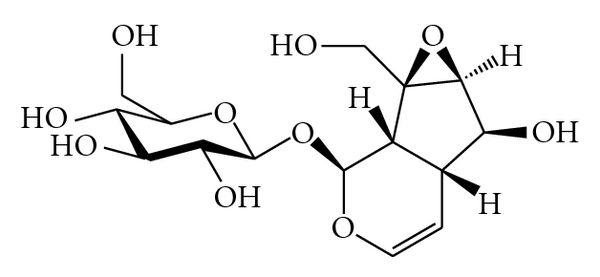
Chemical structure of catalpol.

 Recently, catalpol has been identified as a vital active with robust cognitive potential (Figure 4). Behaviour studies exhibited that catalpol reversed brain damage and memory deficits in mice induced by lipopolysaccharide (LPS) and D-galactose and in gerbils by cerebral ischemia. The nootropic and neuroprotective efficacy of catalpol probably resulted from a variety of underlying molecular mechanisms (Table 7).

- Antioxidant activity: catalpol promoted endogenous antioxidant enzyme activities, superoxide dismutase (SOD) and glutathione peroxidase (GSH-Px), and antioxidant glutathione (GSH), cut down malondialdehyde (MDA) and reactive oxygen species (ROS) generation in PC12 cells and astrocytes primary cultures, exposed to oxygen and glucose deprivation or H_2_O_2_, and in senescent mice induced by D-galactose [79–81, 86, 89, 91, 92].
- Anti-inflammatory activity: catalpol significantly reduced the release of ROS, TNF-*α*, nitric oxide (NO) and inducible nitric oxide synthase (iNOS) expression after A*β* (1–42)-induced microglial activation in primary cortical neuron-glia cultures, and LPS-induced nuclear factor-kappa B (NF-*κ*B) activation in mice [78, 87].
- Neurogenetic activity: catalpol can enhance axonal growth of cortical neurons cultured *in vitro* from 24 h newly born rat, at 1–5 mg/mL and ameliorate age-related presynaptic proteins decline (synaptophysin and GAP-43), and neuroplasticity loss in the hippocampus of the aged rats, by upregulating protein kinase C (PKC) and brain-derived neurotrophic factor (BDNF) [85, 88].
- Antiapoptotic activity: catalpol not only suppressed the downregulation of Bcl-2, upregulation of Bax, and the release of mitochondrial cytochrome c to cytosol, but also attenuated caspase-3 activation, poly-ADP-ribose polymerase (PARP) cleavage, and eventually protected against H_2_O_2_-induced apoptosis in PC12 cells and in the ischemic dorsal hippocampus of gerbils subject to CCAO [82–84, 90].
- In addition, the function to stimulate the production of adrenal cortical hormones, which increases the production of sex hormones, is likely implicated into the cognitive benefit of catalpol in menopausal women [92].

## 4. Discussion and Conclusion

TCM has a long history of human use for mental health. The current literature survey addressing traditional evidence from human studies has been primarily carried out. The top 10 TCM herb ingredients were identified. Poria, thinleaf milkwort, licorice, Chinese Angelica, and Rehmannia were further prioritized to have the highest potential benefit to dementia intervention, due to their highest frequency of use in 236 formulae collected from 29 ancient Pharmacopoeias, ancient formula books, or historical archives on ancient renowned TCM doctors, over the past 10 centuries. 

 In TCM philosophy, AD is assumed to be induced by kidney essence vacuity and toxin (turbid phlegm). The amnestic mild cognitive impairment in elderly population has been disclosed in a clinical investigation to correlate with kidney essence vacuity and turbid phlegm blocking upper orifices. The whole cognitive function may worsen because of the aggravation of kidney essence vacuity, deficiency of blood and *qi*, phlegm and heat toxin and may eventually lead to multiple cognitive domains impairment, even dementia [93].

 Based on the history of use, there is strong clinical support that *Radix polygalae *is memory improving since its efficacy has been demonstrated in elderly with mild cognitive decline [32, 33]. There is suggestive evidence that *Poria cocos*, *Radix glycyrrhizae*, *Radix Angelica sinensis*, or *Radix rehmanniae *are memory improving, though modern clinical reports concerning the four herbs are absent yet. 

 Furthermore, pharmacological investigations in 39 animal studies and 18 *in vitro* studies also indicated that the five ingredients can elicit memory-improving effects via multiple mechanisms of action, covering estrogen-like, cholinergic, antioxidant, anti-inflammatory, antiapoptotic, Neurogenetic, and anti-A*β* activities. These mechanisms are in well accordance with modern pharmacotherapy for AD and VD, by prescribing ChEIs, anti-inflammatory mediations, antioxidants, estrogen, neurotrophic factors, and nootropics, depending on difference situations. 

 In the meantime, 11 active molecules have also been identified, including sinapic acid, tenuifolin, isoliquiritigenin, liquiritigenin, glabridin, ferulic acid, Z-ligustilide, N-methyl-beta-carboline-3-carboxamide, coniferyl ferulate and 11-angeloylsenkyunolide F, and catalpol. Most of them are lipophilic compounds with comparatively low-molecular weight (200 ~ 700) and likely to be absorbed into blood and distributed to brain according to Lipinski rule of 5 [94]. The 11 compounds can serve as active markers for characterisation and standardization of corresponding TCM herbal extracts and pharmacokinetics markers for bioavailability study. In drug discovery, these phyto-chemicals can also be used as candidates to optimize derivatives [95]. 

 Taken together, it is concluded that TCM could have a complementary and alternative role in preventing and treating cognitive disorder in the elderly. The scientific evidence is being continuously mined to back up the traditional medical wisdom and product innovation in the healthcare sectors.

## Figures and Tables

**Table 1:** TCM formulae selected from ancient Chinese documents.

Classification	Book name	Dynasty	Formulae amount
Pharmacopoeia	*Sheng Ji Zhong Lu *	Song (10–13 century)	45
	*Tai Ping Hui Min He Ji Ju Fang *	Song (10–13 century)	2
	*Tai Ping Sheng Hui Fang*	Song (10–13 century)	2
	*Pu Ji Fang *	Ming (14–17 century)	2
	*Yi Fang Lei Ju*	Ming (14–17 century)	2
	*Yi Zong Jin Jian *	Qing (17–19 century)	9

Formulae monographs	*Zhou Hou Fang*	Jin (3-4 century)	1
	*Qian Jin Yao Fang *	Tang (7–10 century)	3
	*Ren Zhai Zhi Zhi Fang Lun*	Song (10–13 century)	3
	*Fu Ren Da Quan Liang Fang *	Song (10–13 century)	1
	*Shi Zhai Bai Yi Xuan Fang*	Song (10–13 century)	5
	*Shi Yi De Jiu Fang*	Yuan (13-14 century)	4
	*Qi Xiao Liang Fang*	Ming (14–17 century)	29
	*Gu Jin Yi Jian*	Ming (14–17 century)	1
	*She Sheng Zhong Miao Fang*	Ming (14–17 century)	1
	* Zheng Zhi Bao Jian*	Qing (17–19 century)	1
	*Ji Yan Liang Fang*	Qing (17–19 century)	4

Medical edition	*Yan Yonghe's medical edition*	Song (10–13 century)	13
	*Chen Wuze's medical edition *	Song (10–13 century)	9
	*Dan Xi Xin Fa *	Yuan (13-14 century)	4
	*Shou Shi Bao Yuan*	Ming (14–17 century)	21
	*Jing Yue Quan Shu*	Ming (14–17 century)	21
	*Zheng Ti Lei Yao*	Ming (14–17 century)	2
	*Lei Zheng Zhi Chai *	Qing (17–19 century)	16
	*Bian Zheng Lu*	Qing (17–19 century)	16
	*Zha Bing Yuan Liu Xi Zhu *	Qing (17–19 century)	2
	*Yi Xue Zhong Zhong Can Xi Lu*	Modern (20 century)	7

Sum			236

**Table 2:** Top 10 memory-improving TCM herbs.

Chi name	English name	Latin name	Part	Plant	Frequency
Fu Ling	Poria	*Poria cocos*	Sclerotium	*Poria cocos *(Schw.) Wolf	182
Ren Shen	Ginseng	*Radix et rhizoma ginseng*	Root, stem	*Panax ginseng* C. A. Mey.	169
Yuan Zhi	Thinleaf milkwort	*Radix polygalae*	Root	*Polygala tenuifolia *willd. *Polygala sibirica *L.	139
Gan Cao	Licorice	*Radix et rhizoma glycyrrhizae*	Root, stem	*Glycyrrhiza inflata* Bat. *Glycyrrhiza uralensis *Fisch. *Glycyrrhiza grabra* L.	100
Dang Gui	Chinese Angelica	*Radix Angelica sinensis*	Root	*Angelica sinensis* (Oliv.) Diels	84
Shi Chang Pu	Grassleaf sweelflag rhizome	*Rhizoma acori tatarinowii*	Stem	*Acorus tatarinowii *Schott.	80
Suan Zao Ren	Spina date seed	*Semen ziziphi spinosae *	Seed	*Ziziphus jujuba Mill.var.spinosa. *(Bunge) Hu ex H.F. Chou	79
Shu Di Huang	Prepared rehmannia root	*Radix rehmanniae *	Root	*Rehmannia glutinosa* Libosch.	62
Mai Dong	Dwarf lilyturf tuber	*Radix ophiopogonis*	Root	*Ophiopogon japonicus *(L.f.) Ker-Gawl.	62
Sheng Jiang	Fresh ginger	*Rhizoma zingiberis*	Stem	*Zingiber officinale* Rosc.	53

(Note: data are cited from Pharmacopoeia of PR China 2005).

**Table 3:** Memory-improving and neuro-protective effects of *Poria cocos. *

Test	Test materials/dose	Test model	Endpoints/biomarkers	Effects	Reference
*In vivo *	Extracts 20–100 mg/kg	Scopolamine-treated rats	Eight-arm radial maze	Improve spatial memory	[20]
	Extracts 250–500 mg/kg	Innate rats	Electro-physiology Spike amplitude	Enhance hippocampal LTP	[21]
	Methanol extracts 200 mg/mL	Ellman ChE	ChE activity	Inhibit ChE by 27.8%	[22]
	Aqueous extracts 0.2 mg/mL	Innate ICR mice	AChE activity	Inhibit AChE by 13.9%	[23]

*In vitro*	Aqueous extracts 31–250 *μ*g/mL	Brain neurons–neonatal rats	Cytosolic [Ca^2+^] i	Regulate bi-directly [Ca^2+^] i	[24]

Long-term potentiation (LTP); choline esterase (ChE)*; *acetylcholinesterase (AChE).

**Table:** (a) Memory-improving and neuro-protective effects of *Radix polygalae *

Test	Test materials/dose	Test model	Endpoint/biomarkers	Effects	Mechanisms	Reference
Clinic	Extracts 300 mg/d, 4 w	Healthy Korean elderly with subjective memory impairment and mild cognitive impairmentdouble-blind, placebo-controlled, randomized, parallel study	Korean version of California verbal learning testSelf-ordered pointing test	Improve verbal memory No adverse events, except mild dyspepsia	N.A.	[32, 33]

*In vivo *	Extracts i.p., 2 mg/kg	Innate rats	Nestin/BrdU Tuj1/BrdU	Improve memory Promote neuro-genesis	Promote proliferation Promote neurite outgrowth	[34]
	Extracts	Stress-treated rats	Glucose utilization Cell adhesion molecule	Improve memory	Increase glucose utilization Increase total NCAM	[35]
	Extracts 2 g/kg, 1–3 w	NBM-lesioning rats	Neurological test Step-through test	Improve memory	N.A.	[36]
	Extracts i.p., 10 mg/kg	Scopolamine-treated rats	Passive avoidance test water maze test AChE	Improve memory	Inhibit AChE	[36]

*In vitro *	Extracts 0.5–5 *μ*g/mL	Rat primary neurons exposed to Glutamate or A*β*	Cell viability	Protect neurons	N.A.	[37]
	Extracts 0.05–5 *μ*g/mL	Rat cerebellar granule neurons exposed to NMDA	Glutamate release (Ca2+)i/ROS	Protect neurons	N.A.	[38]
	Extracts 0.1–100 *μ*g/mL	Rat cortical neurons exposed to A*β* 25–35	Axonal length Neuro-filament-H/MAP-2Cell viability	Activate axonal extension Protect neurons	N.A.	[39]

Acetylcholinesterase (AChE); bromodeoxyuridine (BrdU); microtubule-associated protein-2 (MAP-2); nucleus basalis magnocellularis (NBM); neural cell adhesion molecule (NCAM); N-methyl-D-aspartic acid (NMDA); reactive oxygen species (ROS); *β* amyloid (A*β*); not available (N.A.); intraperitoneally (ip.).

**Table:** (b) Memory-improving and neuro-protective effects of active components from *Radix polygalae *

Test	Test materials/dose	Test model	Endpoints/biomarkers	Effects	Mechanisms	Reference
*In vivo *	Sinapic acid 10–100 mg/kg	Scopolamine-treated rats	Radial maze test	Improve memory	N.A.	[42, 43]
	Sinapic acid 3–100 mg/kg, 1 h	Scopolamine-treat mice Basal forebrain lesioning mice	Step-through test Ach/ChAT	Improve memory	N.A.	[49]
	Tenuifolin 20–80 mg/kg, 15 d	Aged mice Dysmnesia mice	Step-down test Y maze trial AChE,NE,DA,5-HT	Improve memory	Increase NE and DA Inhibit AChE	[45]
	Tenuigenin 18.5–74 mg/kg	A*β* 1-40-treated rats ibotenic acid-treated rats	Step-through test AchE, ChAT	Improve memory	Cholinergic	[46]
	Acylated oligosaccharides1–10 mg/kg	Scopolamine-treated rats	Step-through test	Improve memory	Cholinergic	[44]

*In vitro *	Tenuigenin 1–4 *μ*g/mL	APP-transfected SH-SY5Y cells	Fluorescence resonance energy transfer	Inhibit A*β* secretion	Inhibit BACE1	[47]
	Onjisaponin 10 *μ*M	Serum deficiency or glutamate-treated PC12 cells	Cell survival	Protect PC 12 cells	N.A.	[48]

Acetylcholine (Ach); acetylcholinesterase (AChE); *choline* acetyltransferase (ChAT); 5-hydroxytryptamine (5-HT); dopamine (DA); norepinephrine (NE); beta-site APP cleaving enzyme (BACE); amyloid precursor protein (APP); *β* amyloid (A*β*); not available (N.A.).

**Table 5:** Memory-improving and neuro-protective effects of *Radix et rhizoma glycyrrhizae. *

Test	Test materials /dose	Test model	Endpoint/biomarkers	Effects	Mechanisms	Reference
*In vivo *	Extracts 75–300 mg/kg, 7d diet 0.5 or 1%, 6w	Diazepam treated mice	Elevated plus-maze test	Improve memory	Cholinergic	[50]
		Scopolamine treated mice	passive avoidance test			[51]
		A*β* 25–35 treated mice	passive avoidance testMorris water-maze test TBARS/Catalase/AChE	Improve memory	Quench oxidative stress Inhibit AChE	[52]
	Aqueous extracts 150 mg/kg, 7d n-hexane extracts 5 mg/kg, 3d	Innate mice	AChE	Inhibit AChE	N.A.	[53]
	Methanol extract 50–100 mg/kg, 21d	IR treated Mongolian gerbils	Cu, Zn-SOD1CA1 pyramidal cells	Protect neurons	Restore Cu, Zn-SOD1	[54]
	Liquiritigenin 2.3–21 mg/kg, 7d	A*β* (25–35)-treated rats	Morris water maze testReference memory taskProbe taskTwo-way shuttle avoidance taskMAP, Nissle, Notch-2			[55]

*In vivo *	Isoliquiritigenin 5–20 mg/kg, 7d	MCAO-treated rats	MDASOD,GSH-Px, CatalaseNa^+^-K^+^-ATPase, ATPEnergy charge, total adenine nucleotides	Protect brain	Promote energy metabolismInhibit oxidative stress	[56]
	Glabridin 1–4 mg/kg, 3d	Innate Mice	ChE	Improve memory	Inhibit ChE	[57]
	Glabridin 5–25 mg/kg	IR-treated rats Staurosporine-treated neurons	MDA, GSH and SODBax, caspase-3,bcl-2	Protect neurons	Inhibit apoptosisInhibit oxidative stress	[58]

Acetylcholinesterase (AChE); cholinesterase (ChE); thiobarbituric acid-reactive substances (TBARS); superoxide dismutase (SOD); malondialdehyde (MDA); glutathione (GSH); microtubule-associated protein (MAP) 2; middle cerebral artery occlusion (MCAO); *β* amyloid (A*β*); Ischemia-reperfusion (IR); not available (N.A.).

**Table:** (a) Memory-improving and neuro-protective effects of *Radix Angelica sinensis *

Test	Test materials/dose	Test model	Endpoint/biomarkers	Effects	Mechanisms	Reference
*In vivo*	Extracts 1 g/kg	scopolamine-treated rats cycloheximide-treated rats	Step-through test	Improve memory	N.A.	[62]
*In vitro*	Extracts 25–200 *μ*g/ml	A**β*-*treated Neuro 2A cells	MTT assay/Δ*Ψ*m ROS/LPO/GSH	Protect neurons	Quench oxidative stress	[63]

3-(4,5-dimethylthiazol-2yl)-2,5-diphenyltetrazolium bromide (MTT); Lipid peroxidation (LPO); mitochondrial transmembrane potential (Δ*Ψ*m); *β* amyloid (A*β*); glutathione (GSH); not available (N.A.).

**Table:** (b) Memory-improving and neuro-protective effects of active components from *Radix Angelica sinensis *

Test	Test materials/dose	Test model	Endpoint/biomarkers	Effects	Mechanisms	Reference
*In vivo *	Ferulic acid s.c., 5 mg/kg/d, 6 d	dl-buthionine-(S,R)-Sulfoximine treated mice	Object recognition test Oxidative carbonyl protein	Improve memory	Elevate carbonyl protein	[66]
	Ferulic acid 28 d	Trimethyltin-treated mice	Y-maze testPassive avoidance testChAT	Improve memory	Activate ChAT	[67]
	Ferulic acid i.p., 20–80 mg/kg, 3 d	Glutamate-treated mice	Behavioral test histopathology [(3)H]-labeled glutamate bcl-2/caspase-3	Protect brain	NMDA receptor antagonist	[68]
	Ferulic acid 0.006%, 4 w	A*β*1-42-treated mice	Step-through test Y-maze test Water maze test GFAP/IL-1 *β*	Improve memoryProtect brain	Suppress astrocytes immunoreactivities	[69]
	Ferulic acid 50–100 mg/kg	Scopolamine-treated rats Cycloheximide-treated rats	Step-through test	Improve memory	Cholinergic Enhance CBF	[70]
	Z-ligustilide 10–40 mg/kg, 4 w	CCAO-treated rats	Morris water maze Neurons/astrocytes count MDA/SOD/ChAT/AChE	Improve memory	Inhibit oxidative stress Cholinergic	[71]
	Z-ligustilide 20–80 mg/kg	MCAO-treated rats	TTC staining Brain swelling Behavioural score	Protect brain	N.A.	[72]
	Z-ligustilide 5–20 mg/kg	IR-treated ICR mice	TTC staining MDA/GSH-Px/SOD Bcl-2/Bax/caspase-3	Protect brain	Inhibit oxidative stress Inhibit apoptosis	[73]

*Choline* acetyltransferase (ChAT); cerebral blood flow (CBF); glial fibrillary acidic protein (GFAP); interleukin-1 (IL-1); *g*lutathione peroxidase (GSH-PX)*; *2,3,5-triphenyltetrazolium chloride (TTC); subcutaneously (s.c.); ischemia-reperfusion (IR); superoxide dismutase (SOD); malondialdehyde (MDA); acetylcholinesterase (AChE); common carotid arteries occlusion (CCAO); middle cerebral artery occlusion (MCAO); *β* amyloid (A*β*); N-methyl-D-aspartate (NMDA); not available (N.A.).

**Table 7:** Memory-improving and neuro-protective effects of *Radix rehmanniae*.

Test	Test materials/dose	Test model	Endpoint/biomarkers	Effects	Mechanisms	Reference
*In vivo *	Extracts 4.5–9.0 g/kg	MSG-treated rats	Morris maze test Step-down test c-fos, NGF expression	Improve memory	Motivate hippocampal c-fos /NGF expression	[75]
	Extracts 4.5–9.0 g/kg	MSG-treated rats	Morris maze test Step-down test NMDA-R1, GABA-R Glutamine, GABA levels	Improve memory	Motivate hippocampal NMDA-R1/GABA-R expression adjust Glutamine/GABA levels	[77]

*In vitro*	Extracts 0.1–1.0 mg/mL, 1–3 d	C6 glioblastoma cells	GDNF gene expression	Stimulate GDNF expression	Up-regulate cPKC/ERK1/2 pathways	[76]

*In vivo *	Catalpol i.p., 10 mg/kg, 10 d	LPS-treated mice	MMPNF-*κ*B	Improve memoryInhibit inflammation	Inhibit NF-*κ*B activation protect mitochondrial function	[78]
	Catalpol 2.5–10 mg/kg, 2 w	D-galactose-treated mice	Passive avoidance test LDH, GSH-ST, GS, CK	Improve memory	Inhibit oxidative stress Maintain energy metabolism	[79–81]
	Catalpol i.p., 1–10 mg/kg	IR-treated Gerbils	Bcl-2, Bax, NO	Protect CA1 neuronsImprove memory	Inhibit apoptosis Inhibit oxidative stress	[82–84]
	Catalpol i.p., 5 mg/kg, 10 d	Aged rats	GAP-43/synaptophysin PKC, BDNF	Protect neuroplasticity	Up-regulate PKC and BDNF (hippocampus)	[85]

*In vitro *	Catalpol 0.5 mM, 1 h	MPTP-treated neurons	Cells Viability, MAO-B, ROS, MCI, MMP, MPT	Protect neurons	Protect mitochondriaMaintain MAO-B activity	[86]
	Catalpol 0.5 mM, 30 min	A*β*1-42-treated Cortical neurons-glia	Cells Viability TNF-*α*, iNOS, NO, ROS	Protect neurons	Inhibit inflammation	[87]
	Catalpol 0.25–5 mg/ml	Primary rat cortical neurons	Cells Viability NF-200 antigen	Enhance axonal growthNo impact on survival	N.A.	[88]
	Catalpol 0.1–100 *μ*g/ml	OGD-treated PC12 cells	Bcl-2, caspase-3/MMP SOD, GSH-Px	Inhibit apoptosis	Retain Bcl-2 and MMP suppress caspase-3 activation maintain SOD and GSH-Px	[89]
	Catalpol 0.1–1.0 mM	H_2_O_2_-treated PC12 cells	Bcl-2 cytochrome c caspase	Protect neuronsInhibit apoptosis	Prevent cytochrome c release Inactivate caspase cascade	[90]
	Catalpol 0.05–0.5 mM	H_2_O_2_-treated astrocytes	Cells Viability ROS	Inhibit oxidative stress	maintain glutathione Scavenge ROS	[91]
	Catalpol 0.3–275.9 *μ*M, 24 h	OGD-treated mice astrocytes	Cell survival/MMP ROS, NO, iNOS, MDASOD, GSH-Px, GSH	Protect astrocytes	Inhibit oxidative stress	[92]

*Nerve growth factor (NGF); *oxygen-glucose deprivation (OGD); lactate dehydrogenase (LDH); glutathione S-transferase (GSH-ST); glutamine synthetase (GS); creatine kinase (CK); mitochondrial complex I (MCI); mitochondrial membrain potential (MMP); mitochondrial permeability transition (MPT); brain-derived neurotrophic factor (BDNF); *γ*-amiobutyic acid (GABA); lactate dehydrogenase (LDH); nitric oxide (NO); inducible nitric oxide synthase (iNOS); nuclear factor-kappa B (NF-*κ*B); protein kinase C (PKC); 1-methyl-4-phenyl-1,2,3,6-tetrahydropyridine (MPTP); monosodium glutamate (MSG); lipopolysaccharide (LPS); ischemia-reperfusion (IR); monoamine oxidase (MAO); tumour necrosis factor (TNF)-*α*; reactive oxygen species (ROS); superoxide dismutase (SOD); malondialdehyde (MDA); glutathione (GSH); glutathione peroxidase (GSH-Px); glial cell-derived neurotrophic factor (GDNF).
